# *In situ* nanoencapsulation of essential oils in alginate on a cotton fabric for a controlled release system: statistical evaluation, application, and release mechanism

**DOI:** 10.1039/d6ra01501h

**Published:** 2026-07-03

**Authors:** Soraya Ghayempour, Seyed Mahmoud Tabatabaei, Maryam Torabi

**Affiliations:** a Department of Textile Engineering, Yazd University Yazd Iran s.ghayempour@yazd.ac.ir; b Department of Architecture and Art, Science and Arts University Yazd Iran

## Abstract

The application of natural herbal products to the surface of textiles is a safe and novel approach to treating diseases. To achieve a textile-based delivery system for controlled release, the active ingredients can be encapsulated in a natural polymer on the textile. The present study aims to control the release of essential oils through their *in situ* nano-encapsulation into an alginate biopolymer on a cellulosic cotton fabric for managing children's anorexia. For this purpose, cellulosic cotton aprons were finished using three essential oils extracted from peppermint, cardamom, and ginger in the presence of an alginate biopolymer, and analyzed using a statistical assay to select a more effective essential oil as an appetite stimulant. Then, peppermint oil, as the more effective essential oil was *in situ* nano-encapsulated into the alginate biopolymer on the surface of a cellulosic cotton fabric using an ultrasound-based encapsulation method. FESEM images indicated that alginate nanocapsules were successfully synthesized with a diameter of about 70–90 nm and well stabilized on the surface of the cotton fabric. UV-vis spectroscopy and antimicrobial activity assays were used to evaluate the release behaviour of peppermint oil from alginate nanocapsules stabilized on the cellulosic cotton fabric, showing a relatively good controlled release for 60 days. Also, the finished cotton fabric using alginate nanocapsules containing peppermint oil showed no skin sensitivity in rats for 72 h. This study provides a good prospect for future studies on drug delivery systems with the ability to store plant extracts or essential oils for a long time and control their release for the treatment of diseases without side effects.

## Abstract 2

In this study, a controlled release system for managing children's anorexia was prepared through the *in situ* nano-encapsulation of essential oils into an alginate biopolymer on a cellulosic cotton fabric. The graphical abstract was partially generated with Google Gemini.

## Graphical abstract

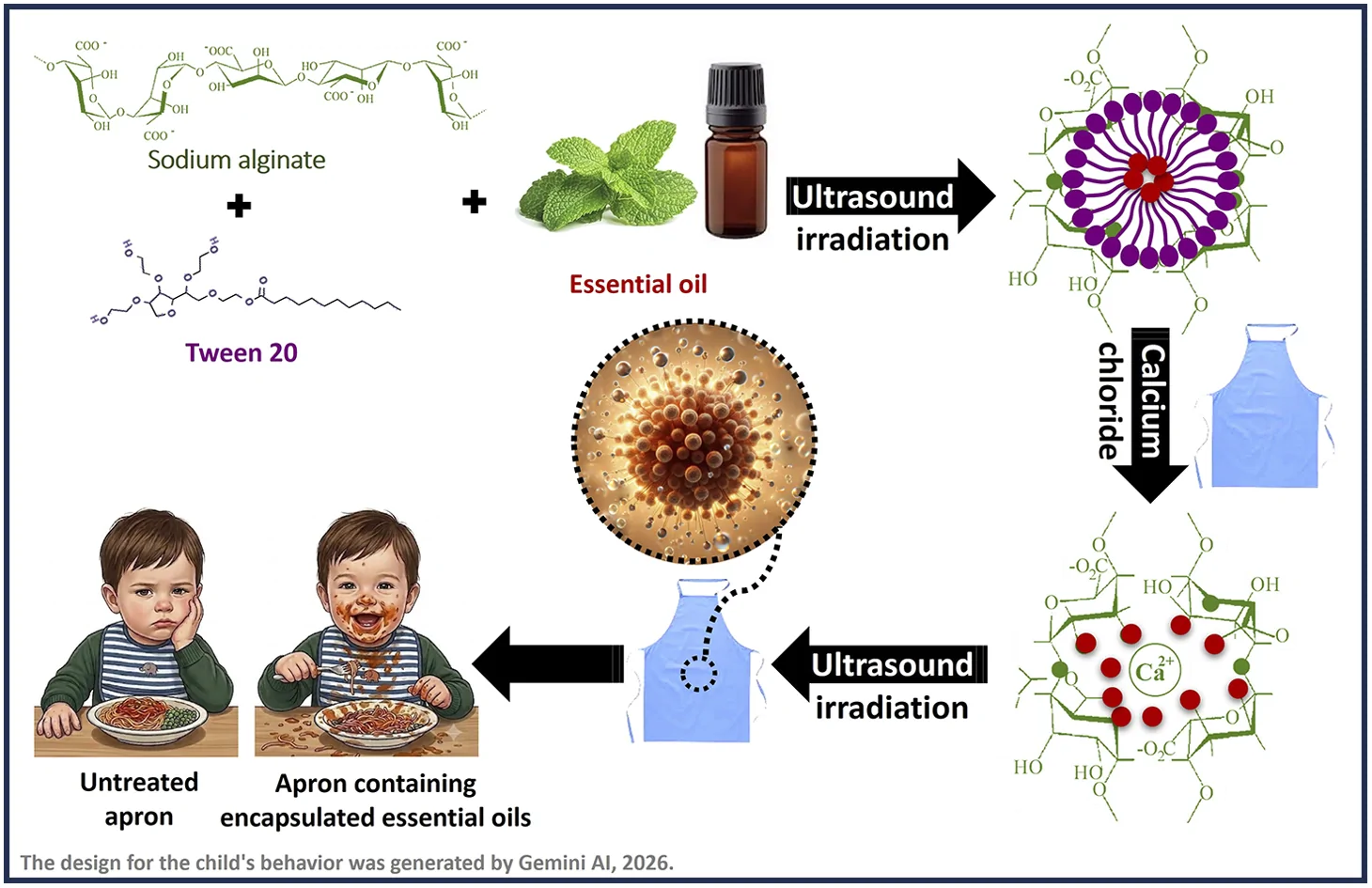

## Introduction

Nanotechnology, one of the impactful technologies of the 21st century, is the science of the synthesis and control of materials and devices at the nanoscale (1–100 nm). The research area of nanotechnology has been continuously expanding in various fields, such as natural sciences, physics, chemistry, biology, engineering, materials science, food industry, medicine, agriculture, information technology, textiles, and others. Also, green synthesis has been utilized as a low-cost and eco-friendly approach to develop advanced nanostructures.^1–3^ One of the most important methods for producing nanostructures is enclosing a core material inside a nanoscale membrane, called “nanoencapsulation”. This technique is widely applied in various fields, such as food industry, drug delivery systems, biotechnology, and smart textiles. One of the applications of this method is the encapsulation of herbal extracts and natural essential oils. The applications of these useful materials are difficult due to their instability under exposure to environmental factors such as air, light, humidity, and thermal conditions as well as a lack of control over the amount and duration of consumption. Therefore, to obtain an ideal controlled release system, they should be encapsulated in a polymeric wall material.^4,5^

Due to the unique features of alginate, such as good mechanical and physical properties, biodegradability, biocompatibility, and the capability for drug loading, it is a promising wall material for the encapsulation of essential oils and plant extracts. The structure of this biopolymer is composed of the linear copolymers of 1–4 glycosidically linked β-d-mannuronic acid and α-l-guluronic acid residues. Therefore, it can interact with divalent cations to form a three-dimensional alginate gel network (3DAGN) called the “egg-box” model, which is an appropriate space for the encapsulation and controlled release of volatile substances. However, the properties of the prepared 3DAGN depend on the affinity of alginate with the utilized divalent cation, concentration of the cation, and specifications of alginate such as its molecular size and concentration, the ratio of mannuronic and guluronic acids, and others.^6–8^

Encapsulated extracts and essential oils can be stabilized on the surface of textiles to achieve perfumed, antimicrobial, mosquito-repellent, anti-odor, and medical textiles.^9–11^ A major concern in the stabilization of encapsulated essential oils on textiles is the used finishing methods. The disadvantage of some finishing methods, such as pad-dry-cure, printing, immersion, and others, is the instability of the final products due to the volatility of essential oils. In this method, the wall of micro-/nano-capsules may break due to high temperature and pressure.^12,13^ The proposed approach for this issue is a low-temperature and pressure ultrasound-based process, including the *in situ* nanoencapsulation of essential oils in an alginate shell on the surface of textiles to obtain textiles loaded with nanocapsules containing essential oils.

The novelty of this work is the development of a controlled-release system based on *in situ* nanoencapsulation of essential oils on textiles through combining the statistical evaluation of clinical efficacy with materials science. For this purpose, three natural essential oils extracted from peppermint, cardamom, and ginger were applied on the surface of cellulosic cotton aprons in the presence of an alginate biopolymer, and their effects on reducing anorexia in children aged 2 to 5 years were evaluated using a statistical analysis. Peppermint essential oil, as the essential oil with the greatest effect in reducing anorexia in children, was simultaneously encapsulated into the alginate biopolymer and stabilized on the surface of cotton aprons through an ultrasound-based *in situ* nano-encapsulation method to increase durability and controlled release of the essential oil.

## Experimental

### Chemicals

Sodium alginate was received from Manutex FAV, ISP Alginates, UK. Calcium chloride and polyoxyethylene sorbitan monolaurate (Tween 20) were purchased from Merck Co., Germany. Peppermint, cardamom, and ginger essential oils were purchased from Barij Essential Oil Co. Deionized water was used for the preparation of all solutions. A 100% bleached cotton fabric with 140 g m^−2^ areal density, 20 Nm yarn count, 22 yarn per cm warp, and 25 yarn per cm weft was applied as the substrate in the finishing processes.

### Application of essential oils on the cellulosic cotton aprons

**Fig. 1:**
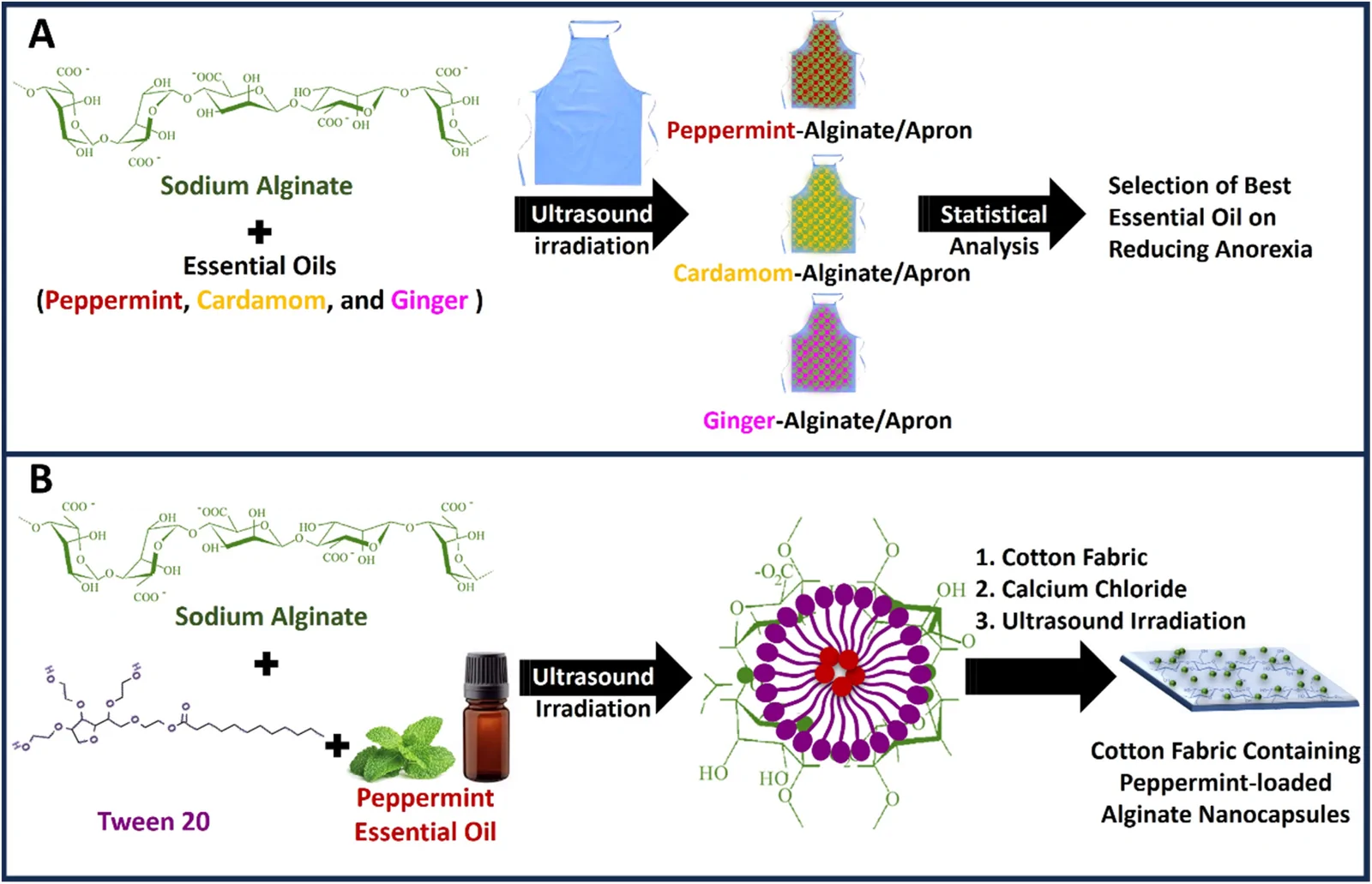
Schematic of the (A) direct application of essential oils and alginate biopolymer on cellulosic aprons and (B) *in situ* nano-encapsulation of essential oils into alginate biopolymer on a cotton fabric.

To select the essential oil with the greatest effect in reducing children's anorexia, firstly, several aprons were designed using a cellulosic cotton fabric. Then, peppermint, cardamom, and ginger essential oils, as the appetite stimulants, were applied on the cotton aprons by the immersion method. The cotton aprons were placed in a solution containing sodium alginate and herbal essential oils and sonicated using a 400 W and 24 kHz ultrasonic homogenizer (UP400S, Hielscher, Germany) for 10 minutes (Fig. 1A). The finished cotton aprons were called peppermint-alginate/apron, cardamom-alginate/apron, and ginger-alginate/apron, and they were used as scented clothings after drying at room temperature. The morphology of the prepared cellulosic cotton aprons containing essential oils was evaluated by FESEM using a Tescan, MIRA3 LM field emission scanning electron microscope.

### Statistical analysis

48 Children present in three kindergartens were selected as the statistical population of the research. The prepared fragrant aprons (peppermint-alginate/apron, cardamom-alginate/apron, and ginger-alginate/apron) were used for 10 days in breakfast and lunch for the children. The weight changes of children were measured in 4 periods of 10 days, without and with aprons containing peppermint, cardamom and ginger essential oils. Also, 70 questionnaires were completed by parents and kindergarten teachers using total population sampling method (examining the entire population). Cronbach's alpha coefficient was used for the evaluation of the questionnaire reliability. After ensuring the reliability of the questionnaire, it was distributed among parents and teachers. The obtained data were collected as frequency distribution tables, graphs, one-sided and two-sided *T*-tests, variance analysis tests, and LSD tests, and used to analyze the research questions using the SPSS software.

### *In situ* nano-encapsulation of essential oils on the cotton fabric

A primary emulsion was prepared by adding 0.1% Tween 20, as the emulsifier, to 0.3 g of essential oil and sonicating using an ultrasonic homogenizer with an amplitude of 100% and 0.5 cycles. A solution containing 1% sodium alginate oil was added to the emulsion and sonication was continued for 10 min. The cellulosic cotton fabric was immersed in the emulsion for 10 minutes. Calcium chloride was added to the mixture, and sonication was continued for 5 minutes until the fragrant nanocapsules formed and were well-stabilized on the surface of the cellulosic cotton fabric. Finally, cotton fabric containing nanocapsules was washed with distilled water to remove excess compounds from the surface. Fig. 1B indicates a schematic for the *in situ* nano-encapsulation of peppermint essential oils into the sodium alginate biopolymer on the cellulosic cotton fabric and its application as a safe and effective treatment method for children's anorexia.

### Characterization of the cotton fabric containing fragrant alginate nanocapsules

The morphology and elemental analysis of the cotton fabric containing fragrant alginate nanocapsules were studied by a FESEM device (VEGA2 TESCAN scanning electron microscope, Czech) coupled with an energy-dispersive X-ray (EDX) device. FT-IR spectra of samples were recorded by a Bruker Equinox 55 single beam spectrometer in the range of 400–4000 cm^−1^ to confirm the presence of alginate nanocapsules on the surface of the finished cotton fabric. The size distribution of the nanocapsules containing essential oils was evaluated using a dynamic light scattering (DLS) device (VASCO KIN, Cordouan Technologies), and their zeta potential was measured using a ZetaSizer analyzer (Nano ZS, Malvern Instruments). The encapsulation efficiency (EE) was calculated by eqn (1):1
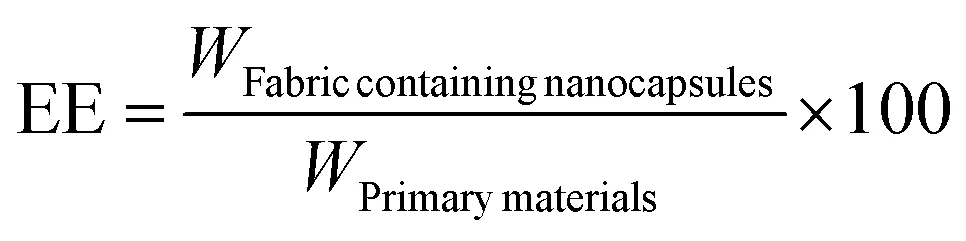
where *W*_Fabric containing nanocapsules_ is the weight of the prepared cotton fabric containing nano-encapsulated essential oil, and *W*_Primary materials_ is the weight of primary materials, including the raw cotton fabric, alginate, essential oil, and calcium chloride.

The release behaviour of essential oils from the alginate nanocapsules stabilized on the cotton fabric was evaluated by an Agilent Cary 100 Ultraviolet-visible (UV-vis) spectrophotometer. For this purpose, one of the aprons used by a child was tested at 10 day intervals. At every stage, the apron was immersed in water and stirred for 30 min. The UV-vis spectra of the solutions were recorded and used to evaluate the release behaviour of essential oils from the cotton fabric containing nanocapsules. The release efficiency (RE) was calculated by eqn (2):2
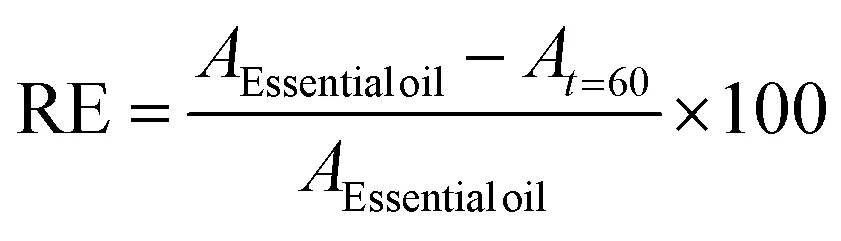
where *A*_Essential oil_ and *A*_*t*=60_ are the absorbance of pure essential oil and the apron containing nanocapsules after 60 days, respectively.

The antimicrobial activity of the cotton fabric containing fragrant alginate nanocapsules was measured using the colony counter method according to the AATCC TM100-2019 standard method. The used microorganisms in the antimicrobial assay include *Escherichia coli* (*E. coli*, ATCC 25923) as the Gram-negative bacteria, *Staphylococcus aureus* (*S. aureus*, ATCC 25922) as the Gram-positive bacteria, and *Candida albicans* (*C. albicans*, ATCC 3153) as the fungus. A colony of microorganisms was inoculated on a nutrient agar plate and cultivated at 37 °C for 24 h. Then, the colonies were inoculated into a 20 mL of broth and cultivated at 37 °C for 24 h. The number of living microorganisms was calculated using the optical density at 580 nm. The microorganism number was adjusted to 1–2 × 10^6^ CFU mL^−1^ using nutrient broth; 1 mL of broth was added to 20 mL of the nutrient culture medium and cultivated at 37 °C for 2 h with shaking. The number of living microorganisms was adjusted to 1 ± 0.3 × 10^5^ CFU mL^−1^. Samples were cut into pieces with a diameter of 48 ± 0.1 mm and sterilized in an autoclave at 121 °C for 15 min. 0.2 mL of the prepared inoculum was applied to the samples and cultivated at 37 °C for 18 h. The percentage of microorganism reduction (*R*) was calculated using eqn (3).3
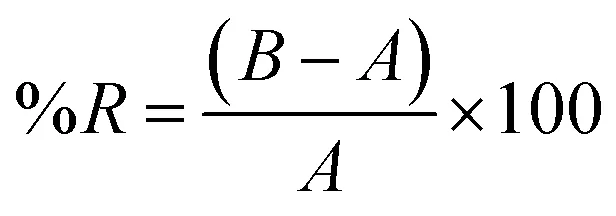
where *A* and *B* are the number of microorganisms on the raw cotton fabric (control) and the cotton fabric containing fragrant alginate nanocapsules, respectively.^14^

Skin sensitivity of the cotton fabric containing fragrant alginate nanocapsules was evaluated through the hypodermic injection of a suspension of alginate nanocapsules containing peppermint oil into a laboratory rat. The appearance of erythema and edema on the skin of the rat was recorded as a sign of skin sensitivity.^4^

The mechanical properties of the cotton fabric containing fragrant alginate nanocapsules including water retention, water absorbency, mass, stiffness, and thickness were studied according to ASTM D2402-07,^15^ AATCC TM79-2010e2(2018)e3,^16^ ASTM D3776/D3776M-20,^17^ ASTM D-1388-64,^18^ and ASTM D1777-96(2019)^19^ standard methods, respectively.

## Results and discussion

The presented work includes two main sections. First, three fragrant cellulosic cotton aprons (peppermint-alginate/apron, cardamom-alginate/apron, and ginger-alginate/apron) were prepared using the immersion method to evaluate their effect on reducing anorexia in children aged 2 to 5 years using a statistical analysis. In the second section, the more effective essential oil was stabilized on the surface of the cotton fabric through an *in situ* nano-encapsulation procedure to obtain a cotton apron with controlled release property.

### Cellulosic cotton aprons containing essential oils

#### FESEM images of cellulosic cotton aprons

**Fig. 2:**
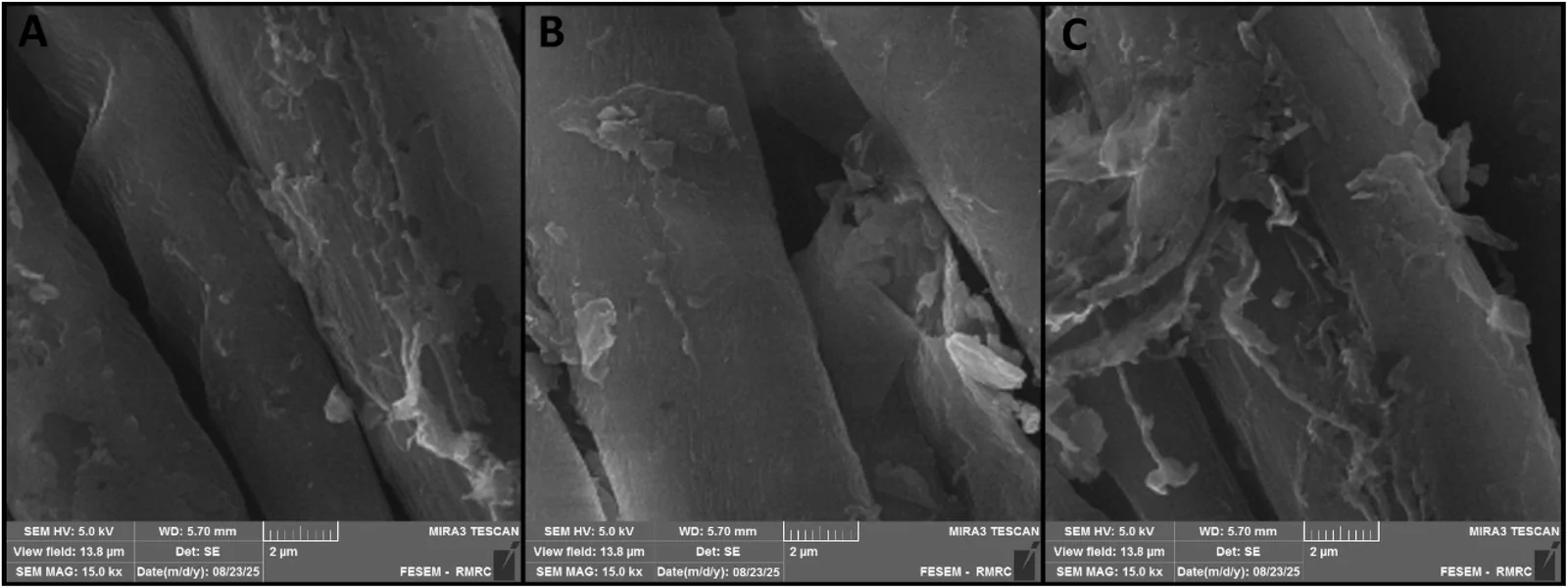
FESEM images of (A) peppermint-alginate/apron, (B) cardamom-alginate/apron, and (C) ginger-alginate/apron.

To investigate the placement of alginate polymers containing various essential oils on the surface of cellulosic aprons, their morphology was examined using FESEM images. As shown in Fig. 2, the alginate biopolymer is stabilized on the surface of cellulose fibres in all three finished samples. The essential oils have likely been trapped within the network-like matrix of alginate. However, the mode of their release is unknown, and it appears that they must be encapsulated in the alginate polymer nanocapsules in the presence of a suitable emulsifier and a coagulant salt to achieve a system with controlled essential oil release.

#### Statistical analysis

The research questionnaire was answered by 70 people, including 48 children's mothers and 22 teachers of kindergartens. Appetite, nausea and abdominal pain, weight, and interest of children were selected as the research variables. The Kolmogorov–Smirnov test was utilized for each of the research variables to choose the appropriate test and evaluate the research hypotheses. As seen in Table S1, since the significance levels of all variables are greater than 0.05, their data distribution is normalized. Therefore, parametric tests were used for data analysis. To statistically examine the effectiveness of essential oils in reducing anorexia, nausea, and abdominal pain in children, eight hypotheses were used, which are listed in the SI. A one-sided *T*-test method was used to confirm or reject the research hypotheses. As shown in Table S2, *P*-values for all hypotheses are less than 0.05. Also, the upper and lower limits of the confidence interval (CI) of 95% are positive for all hypotheses. Therefore, the impact of the variables of hypotheses 1 to 5 on the appetite of children is desirable. The greatness of the average effect of these variables on children's appetite in comparison with the theoretical average indicates that the effect of all these variables is at a high level. Similarly, the effect of variables of hypotheses 6 to 8 on reducing nausea and abdominal pain in children is desirable. Also, the average effect of their variables on the nausea and abdominal pain of children is greater than the theoretical average. Therefore, the impact of all of these variables is at a high level.

#### Effect of the aroma of essential oils on children's appetite, nausea and abdominal pain

The effect of scents on children's appetite, nausea, and abdominal pain was studied using variance analysis. Table S3 indicates that the obtained *P*-values using variance analysis are less than 0.05; therefore, there is a significant difference between the average effects of peppermint-alginate/apron, cardamom-alginate/apron, and ginger-alginate/apron on the appetite, nausea, and abdominal pain of children. In other words, the effect of these scents on the appetite, nausea, abdominal pain, and the interest of children is not the same. Therefore, a two-by-two comparison of scents was done using least significant difference (LSD). As seen in Table S4, the *P*-values of all LSD tests for evaluation of the effect of scents on appetite are less than 0.05. Therefore, there is a significant difference in the average effects of “ginger and peppermint”, “ginger and cardamom”, and “peppermint and cardamom” on children's appetite. On the other hand, the confidence interval between the two variables of ginger and peppermint is negative, so the average effect of ginger scent on children's appetite is lower than that of peppermint scent. The confidence intervals between the two variables of “ginger and cardamom” and “peppermint and cardamom” are positive. Therefore, the average effects of ginger and peppermint scents on children's appetite are more than those of cardamom scent. The study on the effect of scents on nausea and abdominal pain of children indicated that the *P*-values for the LSD tests of ginger and peppermint are more than 0.05. Therefore, the average effect of ginger and peppermint scents on nausea and abdominal pain in children is the same, but there is a significant difference in the average effects of “ginger and cardamom” and “peppermint and cardamom” on nausea and abdominal pain in children. Also, the average effects of ginger and peppermint scents on children's appetite are more than those of the cardamom scent. In the evaluation of children's interest, there is a significant difference between the average level of the interest of children in the smell emitted by ginger-alginate/apron and peppermint-alginate/apron as well as peppermint-alginate/apron and cardamom-alginate/apron. Due to the negative confidence interval between the two variables of ginger and peppermint, the average interest of children to the smell emitted from the ginger-alginate/apron is lower than that of peppermint-alginate/apron, while the average interest of children to the smell emitted from peppermint-alginate/apron is more than that of cardamom due to the positive confidence interval between the two variables of peppermint and cardamom.

#### Children's weight changes

**Fig. 3:**
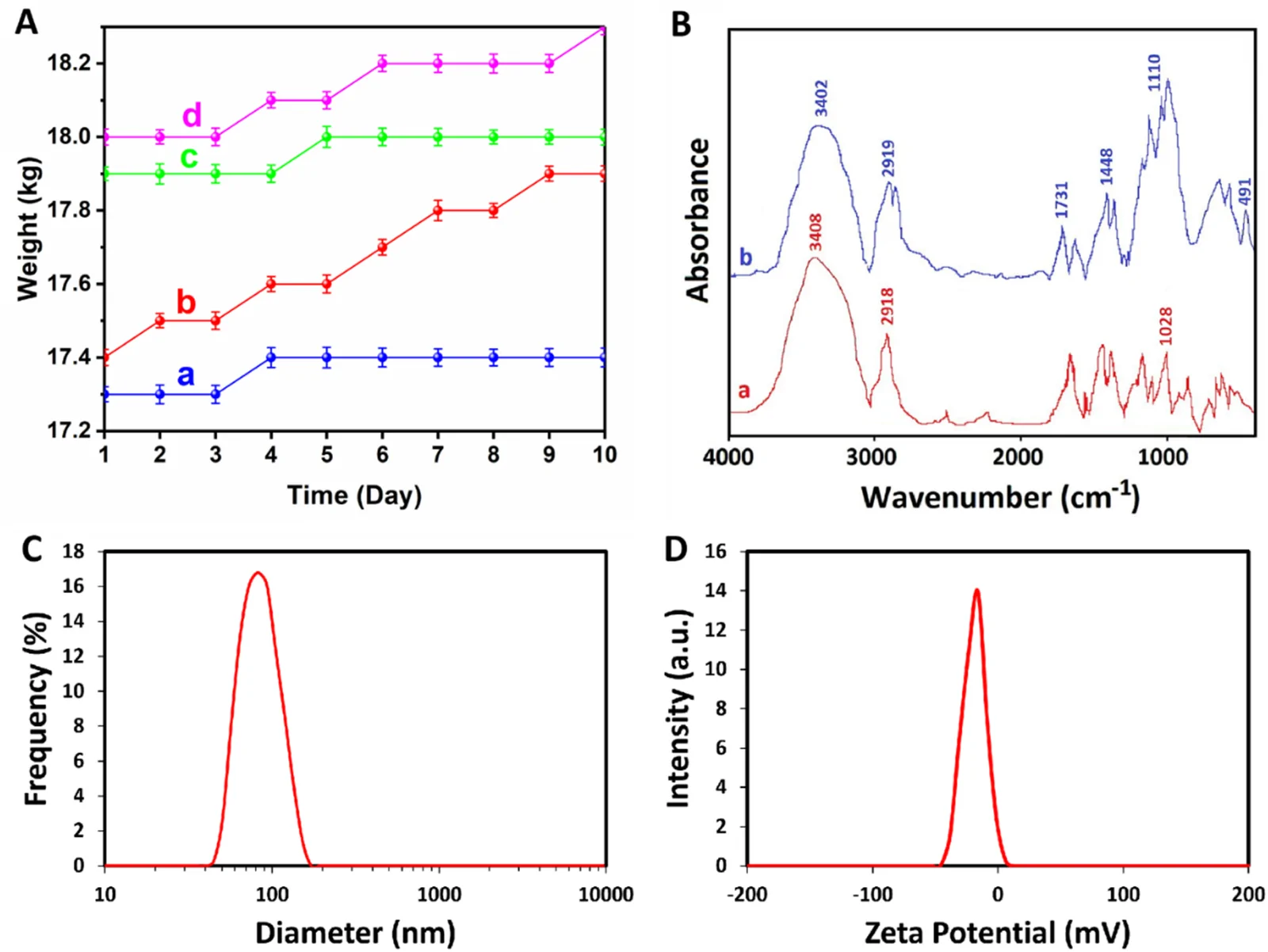
(A) Effect of (a) cellulosic cotton aprons without essential oils and cellulosic cotton aprons containing (b) peppermint-alginate, (c) cardamom-alginate, and (d) ginger-alginate on children's weight changes, (B) FT-IR spectra of the (a) raw cellulosic cotton fabric and (b) cellulosic cotton fabric finished by alginate nanocapsules containing peppermint oil, (C) DLS test of alginate nanocapsules containing peppermint oil, and (D) zeta potential test of alginate nanocapsules containing peppermint oil.

The weight changes in children were investigated by monitoring their weight while they used the finished cotton aprons (peppermint-alginate/apron, cardamom-alginate/apron, and ginger-alginate/apron) during four periods of 10 days. The results of the children's average weight are indicated in Fig. 3A. As can be seen, the greatest effect of weight changes was obtained using the peppermint-alginate/apron. After peppermint, ginger-alginate/apron showed a greater effect on children's weight. Although the effect of cardamom-alginate/apron on children's weight was less than that of the other two essential oil-containing aprons, the weight changes of children using this essential oil were greater than the normal average.

#### *In situ* nano-encapsulation of essential oils on the cotton fabric

The results of statistical analysis indicated a greater effect of peppermint oil in decreasing anorexia in children. Hence, to increase the duration of peppermint oil, it was used in the preparation of a textile-based controlled release system through *in situ* nano-encapsulation of peppermint oil into alginate on the surface of a cellulosic cotton fabric. Another reason to use this method was the inherent instability and volatility of natural essential oils against air, light, humidity, and high temperature.

#### Structure study

Fig. 3B shows the FT-IR spectra of the raw cotton fabric and cotton fabric containing fragrant alginate nanocapsules. The peaks related to stretching vibrations of the hydroxyl groups and C–O–C bonds at 3408 and 1028 cm^−1^ are the important peaks in the spectrum of the raw cotton fabric, respectively.^20,21^ Also, the observed peak at 2918 cm^−1^ is related to alkane C–H bonds present in the cellulosic structure of the cotton fabric. The peak appeared at 1731 cm^−1^ in the FT-IR spectrum of the finished cotton apron can be attributed to the stretching vibration of the carbonyl groups in the structure of menthone and isomenthone compounds in peppermint essential oil encapsulated into the alginate biopolymer.^22–24^ The increase in the height of the peak at 1110 cm^−1^, related to the stretching vibration of the C–O group, and the displacement of the peak appeared at 1448 cm^−1^ compared to FT-IR spectrum of raw cotton fabric confirm the existence of encapsulated peppermint oil on the surface of the finished cotton fabric.^25,26^ The appearance of a peak at 491 cm^−1^ is related to the Ca–O bond, which proves the successful formation of alginate nanocapsules on the surface of the cotton fabric.^21,27^

#### Particle size and zeta potential

Fig. 3C indicates the results of the DLS test of nanocapsules containing essential oils. The average particle size was calculated to be 82.3 nm using this graph, and the hydrodynamic diameter of nanocapsules was between 45 and 151 nm. The zeta potential test was used to measure the colloidal stability and surface charge of nanocapsules containing essential oils. As observed in Fig. 3D, a zeta potential of −15 mV was obtained. The low and negative value of zeta potential can be attributed to the presence of Tween 20 as the emulsifier in the synthesis of nanocapsules.

#### Morphology and elemental analysis

**Fig. 4:**
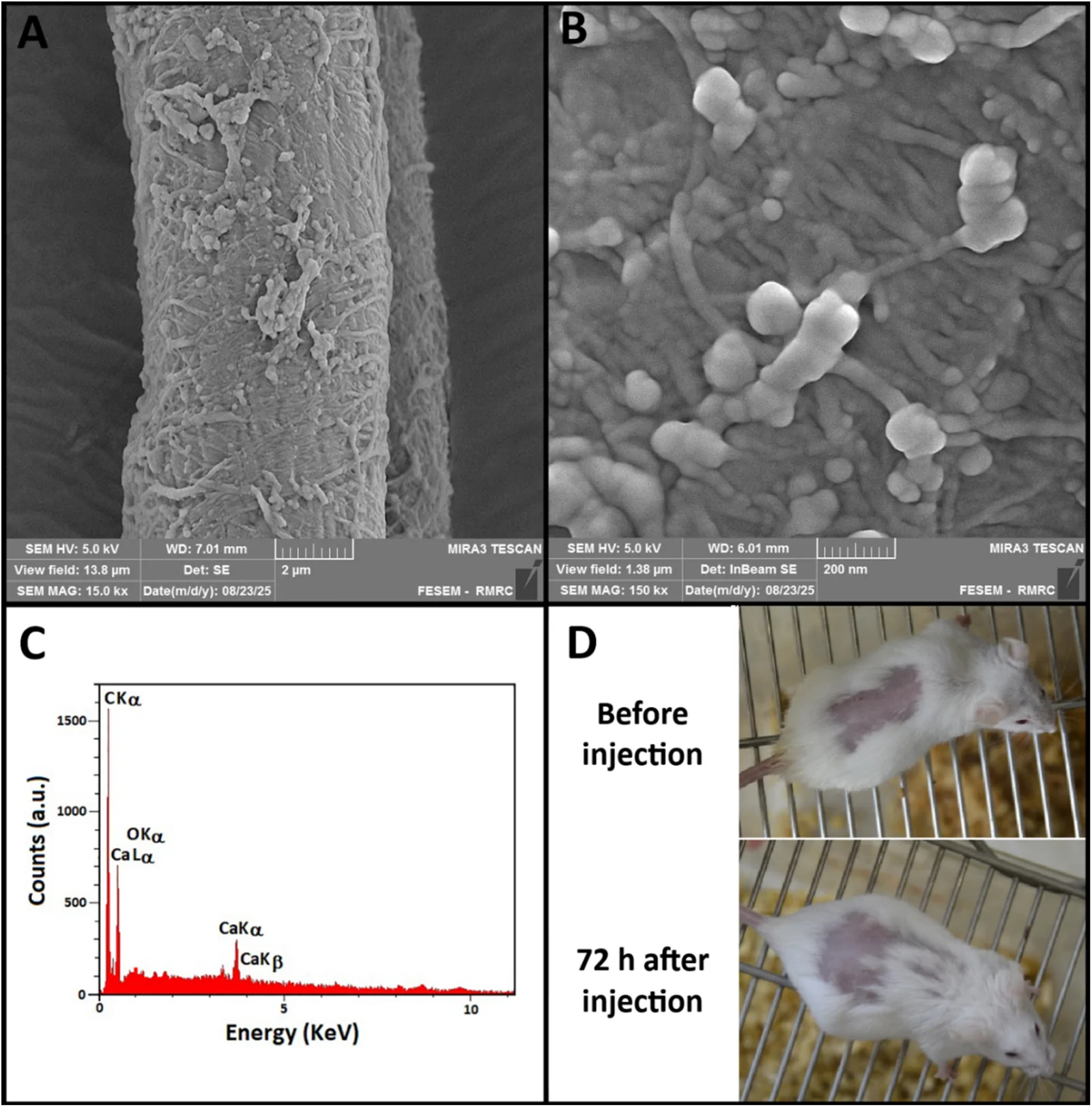
(A and B) FESEM images of the cellulosic cotton fabric finished with alginate nanocapsules containing peppermint oil at two magnifications, (C) EDX pattern of the cotton fabric finished with alginate nanocapsules containing peppermint oil, and (D) appearance of a laboratory rat skin before and 72 hours after the injection of a suspension of alginate nanocapsules containing peppermint oil to assess skin sensitivity.

FESEM images of the cotton fabric finished with alginate nanocapsules containing peppermint essential oil are displayed in Fig. 4A and B. As seen, the alginate nanocapsules are successfully synthesized and stabilized on the surface of cotton fibers with an average size of 70–90 nm. Also, the elemental evaluation of the cotton fabric finished with alginate nanocapsules was carried out by EDX analysis. The appearance of the peaks related to carbon and oxygen (related to the cellulosic cotton fabric and alginate biopolymer) and calcium (as the coagulant reagent) in the EDX pattern of the finished cotton fabric (Fig. 4C) confirms that alginate nanocapsules are successfully anchored on the surface of the cotton fabric.

#### Encapsulation efficiency (EE)

The encapsulation efficiency of the cotton fabric finished with alginate nanocapsules was measured to study the encapsulation performance of peppermint essential oil. An EE of 92.3% was obtained, which indicates the successful encapsulation of peppermint essential oil in alginate biopolymer on the surface of the cotton fabric with a low waste percentage of primary materials. The use of Tween 20 as the emulsifier played an important role in obtaining this EE by creating a protective layer around the particles to prevent aggregation. It is necessary to form a microemulsion with low energy consumption by reducing surface tension.

#### Antimicrobial assay

Antimicrobial activity is an important property of a fabric that comes into direct contact with the skin. Despite the good properties of the cotton fabric, it provides suitable conditions for the growth of microorganisms due to its hydrophilic nature and large surface area, creating a humid environment.^28^ Finishing the cotton fabric with peppermint essential oil as a natural antimicrobial substance can prevent the growth of microorganisms. In this work, due to the volatility of peppermint essential oil, it was encapsulated in the alginate biopolymer and stabilized on the fabric surface for durable antimicrobial properties by controlled release. The antimicrobial activity of the cotton fabric containing alginate nanocapsules was measured according to the AATCC TM100-2019 standard method using *E. coli*, *S. aureus*, and *C. albicans*. The cotton fabric containing alginate nanocapsules demonstrated significant antimicrobial activity, achieving a microbe reduction of 98.1% ± 0.5%, 98.8% ± 0.5%, and 97.5% ± 0.5% against *E. coli*, *S. aureus*, and *C. albicans*, respectively. The antimicrobial activity of the cotton fabric containing alginate nanocapsules can be attributed to the existence of compounds such as α-pinene, d-limonene, eucalyptol, menthone, and isomenthone in the structure of peppermint oil encapsulated into alginate nanocapsules. Peppermint oil released from the alginate wall of nanocapsules prevents the growth of microorganisms on cotton fibers.^29–31^

#### Skin sensitivity assay

The skin sensitivity and irritation of the apron can lead to clinical problems for children. Therefore, evaluation of the skin sensitivity of the exemplary finished cellulosic apron is necessary. Skin sensitivity of the finished cellulosic apron was evaluated *via* the hypodermic injection of a suspension of alginate nanocapsules containing peppermint oil into a laboratory rat. Skin erythema and edema, as signs of skin sensitivity, were investigated before and about 72 hours after the injection. The result of this study shows that the injected solution did not induce any edema or erythema on the rat skin (Fig. 4D). Therefore, the prepared cotton fabric containing nano-encapsulated peppermint essential oil is safe and induces no local irritation or skin sensitivity. The use of natural and non-toxic materials such as alginate biopolymer and peppermint essential oil in the finished cotton fabric has prevented skin sensitivity.^32,33^

#### Mechanical properties

During the nanoencapsulation process, sodium alginate transforms to calcium alginate with a three-dimensional network, encapsulating peppermint oil into its wall. Therefore, it can swell by adsorbing water into its three-dimensional network. On the other hand, due to the cellulosic structure of the cotton fabric, it has a high-water retention ability. As predicted, the stabilization of fragrant alginate nanocapsules on the surface of the cotton fabric led to a 12.2% increase in the fabric's water retention capacity compared to the raw cotton fabric. Water absorbency of the cotton fabric containing alginate nanocapsules increased compared to that of the raw cotton fabric; thus, water was adsorbed onto the finished cotton fabric within less than 1 s due to the hydrogel properties of alginate nanocapsules, while it took 7.8 s for raw cotton fabric. Examining the weight difference of the fabric containing alginate nanocapsules showed a 4.1% increase in weight compared to the raw cotton fabric, which is normal due to the stabilization of alginate nanocapsules containing peppermint essential oil on the cotton fabric surface. The stiffness of cotton fabric containing alginate nanocapsules was 184.1 N m^−1^, with no considerable difference from that of the raw fabric (182.3 N m^−1^). Also, stabilizing nanocapsules on the surface of the cotton fabric led to an insignificant increase (lower than 0.01 mm) in the thickness of the finished fabric against the raw cotton fabric.

#### Release evaluation of peppermint essential oil from the cotton fabric containing alginate nanocapsules

**Fig. 5:**
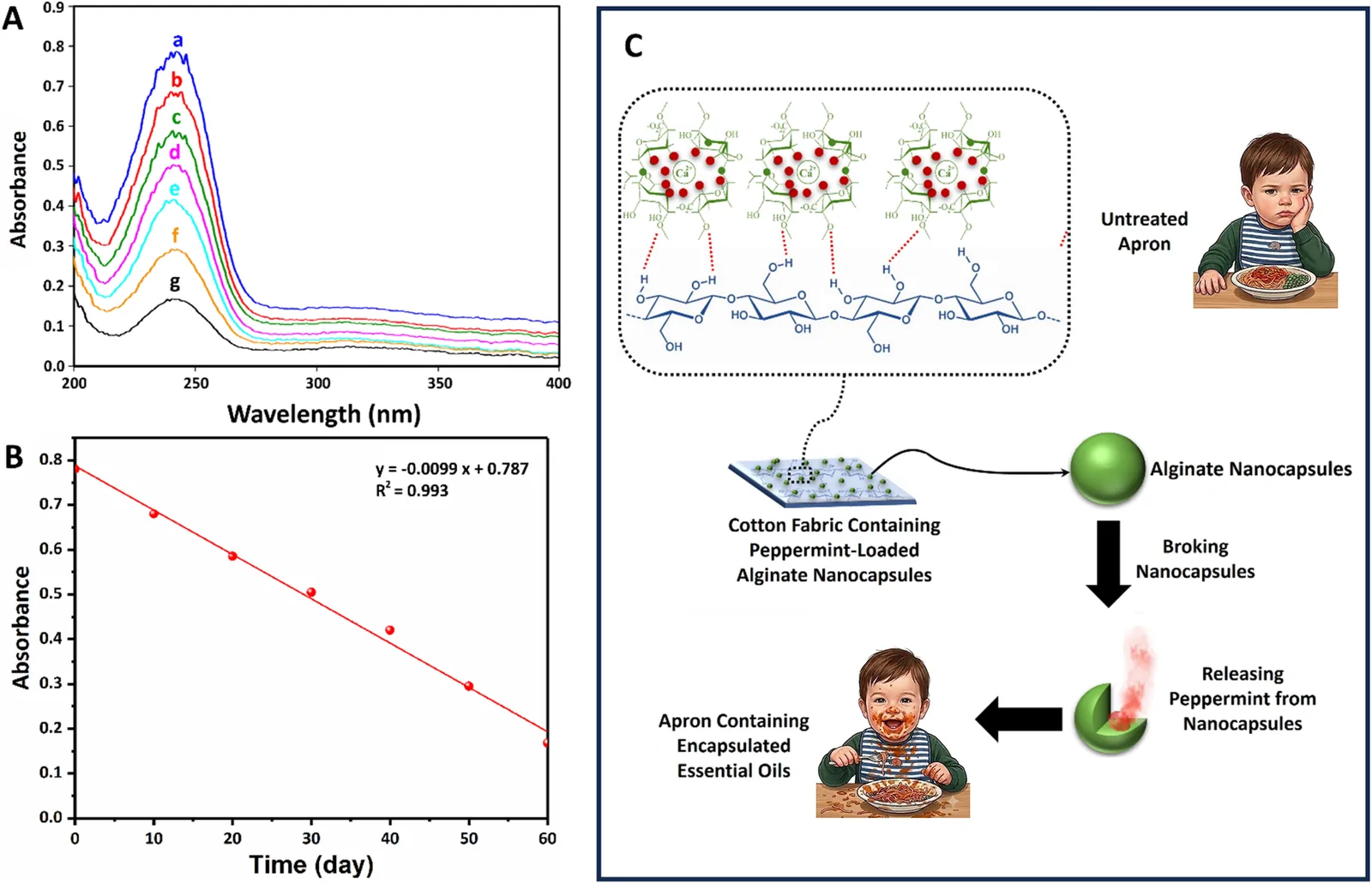
(A) UV-vis spectra of the cotton fabric containing nano-encapsulated peppermint oil after 0, 10, 20, 30, 40, 50 and 60 days (a–g), (B) release kinetics of peppermint oil from alginate nanocapsules on the cotton fabric, and (C) schematic of how encapsulating peppermint oil into alginate biopolymer, and performance of the fabric containing alginate nanocapsules loaded with peppermint oil in reducing anorexia in children. Fig. 5 was partially generated with Google Gemini.

The important advantage of encapsulating essential oils into a polymeric wall on textiles is their controlled release over an extended time. For this purpose, the cotton fabric containing nano-encapsulated peppermint essential oil in alginate biopolymer was used by children for 60 days, and the release of essential oil from the fabric was evaluated at a 10 day interval using UV-vis spectroscopy. The UV-vis spectra of the cotton fabric containing nano-encapsulated peppermint essential oil after 0, 10, 20, 30, 40, 50, and 60 days and the release kinetics of peppermint essential oil from cellulosic cotton fabric are shown in Fig. 5A and B. As observed, the absorbance of samples regularly decreased from 0.79 on the first day to 0.18 on the 60th day. The release efficiency (RE) of peppermint essential oil from the cellulosic cotton fabric was 78.6%, which indicates the good performance of the prepared controlled release system. According to the linear changes in absorbance *versus* time, with an equation of absorbance = −0.0099 time (h) + 0.787; *R*^2^ = 0.993, the cotton fabric containing nano-encapsulated peppermint essential oil exhibited controlled release with zero-order kinetics. This indicated the good performance of the finished cotton fabric in the controlled release of peppermint essential oil. Fig. 5C displays encapsulating peppermint essential oil into alginate biopolymer on the surface of cotton fabric. The synthesized nanocapsules were stabilized on the cotton fabric surface through cross-linking hydrogen bonds between the oxygen elements in the alginate structure and the hydrogen elements in the cellulose structure of the cotton fabric. It also shows the performance of the fabric containing alginate nanocapsules loaded with peppermint oil in reducing anorexia in children. The wall of the alginate nanocapsules stabilized on the surface of the cellulose cotton fabric is broken by rubbing, and the scent of mint essential oil is spread in the air, increasing the child's appetite. Similarly, the release of peppermint oil from the cellulosic cotton fabric was evaluated by measuring its antimicrobial activity at various times. As predicted, the antimicrobial activity of the cotton fabric containing nano-encapsulated peppermint essential oil decreased over 60 days. As observed in Table 1, its antimicrobial activity against *S. aureus*, *E. coli*, and *C. albicans* reached 75.8% ± 0.5%, 76.4% ± 0.5%, and 72.1% ± 0.5% after 60 days, respectively. These results confirm the results of the study of the release behaviour of peppermint oil from cotton fabric using UV-visible spectroscopy. The walls of nanocapsules containing peppermint essential oil placed on the surface of cellulose fabric fibers are destroyed by friction caused by rubbing during the two months of wearing by children, and peppermint essential oil is released from the fabric. This causes the antimicrobial activity of the finished fabric to decrease over time.

**Table 1:** Antimicrobial activity of the cotton fabric finished with alginate nanocapsules containing peppermint oil after 0, 10, 20, 30, 40, 50, and 60 days

Microbe	Antimicrobial activity						
	Time (day)						
	0	10	20	30	40	50	60
*E. coli*	98.1 ± 0.5	95.3 ± 0.5	92.0 ± 0.5	89.5 ± 0.5	85.4 ± 0.5	79.7 ± 0.5	75.8 ± 0.5
*S. aureus*	98.8 ± 0.5	95.5 ± 0.5	92.8 ± 0.5	89.4 ± 0.5	85.7 ± 0.5	80.1 ± 0.5	76.4 ± 0.5
*C. albicans*	97.5 ± 0.5	94.4 ± 0.5	91.1 ± 0.5	88.0 ± 0.5	83.5 ± 0.5	77.1 ± 0.5	72.1 ± 0.5

## Conclusions

This work presents a useful and safe approach for reducing anorexia in children based on the use of natural essential oils on cellulosic cotton aprons. Statistical analysis using the SPSS software was used to investigate the performance of different natural essential oils in reducing children's anorexia. Among three tested essential oils (peppermint, cardamom, and ginger), peppermint essential oil was selected as an effective natural material for reducing children's anorexia; therefore, it was used as the core material in the preparation of alginate nanocapsules on the surface of cotton fabric.

FESEM, EDX, and FT-IR analysis confirmed the successful synthesis and stabilization of nanocapsules containing peppermint essential oil on the surface of the cellulosic cotton fabric. The results of the skin sensitivity assay indicated that the prepared cotton fabric containing nano-encapsulated peppermint essential oil is safe and did not induce any local irritation or skin sensitivity. The release behavior of peppermint essential oil from a cotton apron containing nanocapsules was evaluated using UV-visible spectroscopy and antimicrobial activity during six-time intervals of 10 days, indicating the good performance of the cotton fabric in the controlled release of peppermint essential oil. The prepared antimicrobial, non-sensitizing, safe fabric can be used as a high-performance medical textile in reducing nausea and abdominal pain in children.

## A statement concerning research involving human participants

The work involves the use of children as the human subjects. All children's parents gave their informed consent for inclusion before their children participated in the study.

## Ethical statement

All animal procedures were performed in accordance with the Guidelines for Care and Use of Laboratory Animals of Yazd University and approved by the Animal Ethics Committee of Yazd University.

## Author contributions

Soraya Ghayempour: supervision, methodology, experiment, writing – original draft, writing – review & editing, and analysis. Seyed Mahmoud Tabatabaei: supervision, conceptualization, and validation. Maryam Torabi: experiment, software, and analysis.

## Conflicts of interest

There are no conflicts to declare.

## Supplementary Material

RA-016-D6RA01501H-s001

## Data Availability

The datasets supporting this article, including the results of the Kolmogorov–Smirnov test, the research hypothesis, the one-sided *T*-test, variance analysis, and LSD test, have been uploaded as supplementary information (SI). Supplementary information is available. See DOI: https://doi.org/10.1039/d6ra01501h.
