# Supplementary material for: In situ nanoencapsulation of essential oils in alginate on a cotton fabric for a controlled release system: statistical evaluation, application, and release mechanism
Source: RSC Adv. 2026 Jul 3. Online ahead of print. doi: 10.1039/d6ra01501h (PMC13330783; doi:10.1039/d6ra01501h)
Supplement: RA-OLF-D6RA01501H-s001 [file RA-OLF-D6RA01501H-s001.pdf]

Supplementary Information (SI) for RSC Advances.
This journal is © The Royal Society of Chemistry 2026

Supplementary material

In-Situ Nanoencapsulation of Essential Oils in Alginate on Cotton Fabric for a Controlled Release System: Statistical Evaluation, Application, and Release Mechanism

Soraya Ghayempour*[a], Seyed Mahmoud Tabatabaei[b] and Maryam Torabi[b]

[a]*Department of Textile Engineering, Yazd University, Yazd, Iran*

[b]*Department of Architecture and Art, Science and Arts University, Yazd, Iran*

Table S1. Results of Kolmogorov-Smirnov test for research variables

Variable	Statistic	Sig.[1]
**Appetite**	1.378	0.469
**Nausea and abdominal pain**	1.235	0.255
**Weight**	1.362	0.451
**Interest**	1.092	0.217

[1]Significance level

Research hypotheses

Hypothesis 1: The use of herbal essential oils has an appetizing effect in aroma therapy.

Hypothesis 2: Finishing of children's clothing with plant essential oils is effective in removing their anorexia.

Hypothesis 3: The aroma of peppermint essential oil enhances children's appetite.

Hypothesis 4: The aroma of cardamom essential oil enhances children's appetite.

Hypothesis 5: The aroma of ginger essential oil enhances children's appetite.

Hypothesis 6: The aroma of peppermint essential oil reduces children's nausea and abdominal pain.

Hypothesis 7: The aroma of cardamom essential oil reduces children's nausea and abdominal pain.

Hypothesis 8: The aroma of ginger essential oils reduces children's nausea and abdominal pain.

Table S2. One-sided T-test for hypotheses

Hypothesis	μ[1]	σ[2]	t	ν[3]	P-Value	CI[4]	
						Upper limit	Lower limit
**1**	4.11	0.808	11.5	69	0.000	1.30	0.921
**2**	3.98	0.908	9.07	69	0.000	1.20	0.700
**3**	4.40	1.79	14.9	69	0.000	0.580	1.21
**4**	3.47	1.12	3.50	69	0.001	0.739	0.203
**5**	3.86	1.17	5.93	64	0.000	1.15	0.571
**6**	4.35	0.817	13.9	69	0.001	1.55	1.16
**7**	3.41	1.12	3.08	69	0.003	0.682	0.146
**8**	4.27	0.892	11.5	64	0.000	1.49	1.05

[1]Average

[2]Standard deviation

[3]Degrees of freedom

[4]Confidence interval

Table S3. Effect of scents on appetite, nausea and abdominal pain, and scent of children using variance analysis

Variable	Changes source	SS[1]	v[2]	MSB[3]	F- Value	P-Value
**Appetite**	Between-group	30.40	2.00	15.2	14.10	0.00
	Within-group	218.0	202	1.07		
	Total	248.4	204			
**Nausea and abdominal pain**	Between-group	37.90	2.00	18.9	20.8	0.00
	Within-group	184.1	202	0.91		
	Total	222.0	204			
**Interest**	Between-group	53.29	2.00	26.644	24.3	0.00
	Within-group	225.8	206	1.096		
	Total	279.1	208			

[1]Sum of squares

[2]Degrees of freedom

[3]Mean sum of squares

Table S4. Effect of scents on appetite, nausea and abdominal pain, and interest of children using LSD test

Variable	First scent	Second scent	P-Value	Upper limit	Lower limit
**Appetite**	Ginger	Peppermint	0.003	-0.185	-0.891
	Ginger	Cardamom	0.03	0.742	0.037
	Peppermint	Cardamom	0.00	1.27	0.582
**Nausea and abdominal pain**	Ginger	Peppermint	0.626	0.244	-0.404
	Ginger	Cardamom	0.00	1.18	0.538
	Peppermint	Cardamom	0.00	1.26	0.624
**Interest**	Ginger	Peppermint	0.00	-0.686	-1.386
	Ginger	Cardamom	0.69	0.42	-0.277
	Peppermint	Cardamom	0.00	1.45	0.757
